# Comparison of Shaping Ability and Apical Debris Extrusion Using 4 Different Nickel–Titanium Single-File Systems

**DOI:** 10.1155/ijbm/2161833

**Published:** 2025-07-04

**Authors:** Siyu Li, Mengzhen Tang, Xi Wang, Jian Yang

**Affiliations:** ^1^Department of Cariology and Endodontics, The Affiliated Stomatological Hospital, Jiangxi Medical College, Nanchang University, Nanchang 330006, China; ^2^Jiangxi Provincial Key Laboratory of Oral Diseases, Nanchang 330006, China; ^3^Jiangxi Provincial Clinical Research Center for Oral Diseases, Nanchang 330006, China

**Keywords:** apical debris extrusion, centering ability, Ni–Ti instrumentation, simulated root canals, single-file system

## Abstract

**Background:** This study compared the shaping ability and apical debris extrusion of four nickel–titanium (Ni–Ti) single-file systems in simulated curved root canals.

**Methods:** Forty simulated curved root canals in resin blocks were randomly assigned to four groups (*n* = 10): Reciproc Blue (RCB), V-Blue, One Plex, and S-ONE. Images of the simulated root canals were captured before and after instrumentation. The two layers were processed and superimposed using specialized software. Eleven points (Levels 0–10) were selected at 1-mm intervals starting from the apex for evaluation. The amount of resin removed from both the inner (X1) and outer sides (X2) of the root canal, as well as the final canal width (Y), were measured. The centering ratio was calculated using the formula (X1 − X2)/Y to assess the centering ability of the instruments. Apically extruded debris was collected during the root preparation.

**Results:** The preparation times for the root canal were as follows: One Plex > RCB > V-Blue > S-ONE (*p* < 0.001). All four Ni–Ti files were effective in straightening the root canal, with no significant difference in curvature change (*p* > 0.05). At the apex, One Plex exhibited significantly greater deviation compared with the other three groups (*p* < 0.05). At Levels 7-8, the deviation with RCB was significantly greater than with One Plex and S-ONE (*p* < 0.01). The amount of apical debris extrusion in the One Plex group was significantly higher than that in the others (*p* < 0.01).

**Conclusions:** S-ONE demonstrated the best centering ability compared with other groups. In contrast, One Plex produced the highest amount of apical debris extrusion and exhibited transportation at the apical foramen. At Levels 6–8, RCB exhibited excessive removal of the inner canal wall relative to S-ONE and One Plex.

## 1. Introduction

Root canal treatment is the primary approach for managing diseases of the dental pulp and periapical tissues [1]. The root preparation is pivotal in determining the success of endodontic treatment [2]. Its primary objective is to remove necrotic pulp tissue, infected dentin, residual microorganisms, and their metabolic byproducts from the root canal [3], while shaping the canal into a continuous, tapered form that preserves its original anatomy [4]. This step is essential for subsequent root canal irrigation, disinfection, and filling. However, the root canal system is often anatomically complex and interconnected, with curved canals being particularly prevalent, affecting approximately 84% of the cases [5, 6]. These curved canals pose significant challenges during preparation [7], including issues such as loss of working length, apical opening, canal deviation, and ledge formation [8], which demand instruments with higher performance standards [9]. Nickel–titanium (Ni–Ti) instruments, due to their superior flexibility and resistance to torsional fracture compared with stainless steel instruments [10], are better able to maintain the canal's original shape. The clinical use of Ni–Ti instruments in root canal treatment enhances the efficiency of dental practitioners and reduces the risk of complications, such as ledges and perforations.

To improve flexibility and resistance to cyclic fatigue, manufacturers have introduced innovations in both material composition and instrument design [11], including various heat treatment processes and alterations in cross-section and taper designs. Yared's [12] introduction of the single-file root canal preparation concept revolutionized the process, making it more convenient and efficient while also reducing chairside operation time [13]. Consequently, it is crucial to evaluate the preparation efficacy of various single-file systems in curved root canals to guide clinical practice.

Reciproc Blue (RCB; VDW, Munich, Germany), V-Blue (FengJiang, SuHang, China), and S-ONE (SANI, Chengdu, China) are three single-file systems that operate using reciprocating motion. They undergo repeated heating and cooling cycles to form a titanium oxide layer, which alters the surface color of the alloy based on its thickness [14]. A 60–80 nm thickness results in a blue surface, referred to as Blue-wire, while a 100–140 nm thickness produces a gold surface, termed Gold-wire [15]. This heat fabrication treatment changes the molecular structure of the alloy, endowing the material with higher flexibility and resistance to cyclic fatigue [16]. The alloy transitions from controlled to temperature memory, predominantly maintaining a martensitic phase at room temperature and demonstrating minimal rebound after bending under stress [17]. One Plex (ORODEKA, Jining, China), unlike the others, operates with continuous rotary motion. Made from CM-wire treated with heat, it does not have an oxide layer on the surface.

Research comparing the shaping ability of instruments often employs natural teeth and resin-simulated root canals as models. While natural teeth better reflect real clinical situations, their complex and variable anatomy complicates standardization. Peters [18] posited that anatomical variation in natural teeth significantly impacts research results, often more than the instruments themselves. Given the established correlation between root canal anatomy complexity and preparation quality [19], standardized resin blocks with consistent length, taper, diameter, curvature radius, and hardness offer a more reliable model [20]. Therefore, this study uses resin-simulated root canals to assess the shaping ability and apical debris extrusion of the three reciprocating Ni–Ti systems (RCB, V-Blue, and S-ONE) and the continuous rotary system (One Plex). The null hypothesis tested is that no significant differences exist among the four systems in the preparation of severely curved root canals.

## 2. Materials and Methods

### 2.1. Specimen Grouping and Preparation

In this study, 40 L-shaped resin root canals (Dentsply Maillefer, Ballaigues, Switzerland) were selected for evaluation under a microscope (OPMI S7, Carl Zeiss, Oberkochen, Germany). The inclusion criteria were strictly adhered to, with exclusion applied to resin blocks exhibiting surface scratches or blocked canals. The root canals had a consistent curvature of 30° (Schneider method) [21], with an apical foramen diameter of 0.15 mm, taper of 0.02, and working length of 16 mm. To ensure patency of the apical foramen and to create a straight path, the ISO 10/0.02 and 15/0.02 stainless steel K-files (MANI, INC., Japan) were used to slightly exceed the root length. After cleaning, the root canals were subjected to ultrasonic oscillation (VDW EDDY, Munich, Germany) in distilled water for 5 min, dried with absorbent paper points, and injected with black ink (Winsor and Newton, Colart Tianjin Art Materials, Tianjin, China). To seal the root canals, light-cure flowable resin (Filtek Z350 XT) (3M, USA) was applied at the apical foramen and the orifice. The 40 resin root canals were randomly divided into four groups (*n* = 10 per group): RCB, V-Blue, One Plex, and S-ONE. Each resin root canal was individually numbered and marked. A fixed platform was established to position the resin blocks and a camera (Canon EOS 700D, Japan) at a consistent location to capture preoperative images of the simulated curved root canals.

### 2.2. Debris Collection Device

**Figure 1:**
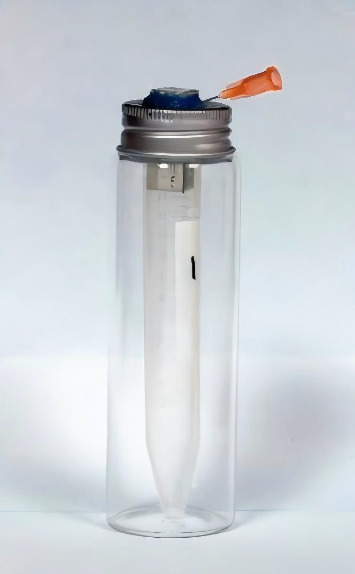
Apically extruded debris collection device.

To prepare the centrifuge tubes for debris collection, 40 15 mL centrifuge tubes were weighed by cap removal, with the average weight recorded three times using a 0.0001 g precision electronic balance (ME204E, METTLER TOLEDO, China), establishing the original weight of the tubes. These were then randomly divided into 4 groups (*n* = 10). In addition, 40 glass bottles, each with a silver aluminum cap and rubber stopper (100 mL capacity), were prepared. The height of the glass bottles matched that of the centrifuge tubes, and after removing the caps, the centrifuge tubes were placed inside the glass bottles. In each bottle's cap and rubber stopper, a square hole of the same diameter as the resin root canal was created. The resin blocks were then inserted through the square holes, with the glass bottle caps tightly sealed. To ensure proper alignment, the contact point between the resin block and the glass bottle cap was bonded with flowable resin. In addition, to equalize internal and external pressure during the root canal preparation, a 5-mL disposable syringe needle was inserted into the glass bottle cap, as shown in Figure 1. The needle's seal was fixed with additional sealant. Throughout the root canal preparation process, each glass bottle was wrapped in tin foil, leaving only the section above the resin root canal orifice visible to eliminate experimental errors from the operator's movements.

### 2.3. Root Canal Preparation

**Figure 2:**
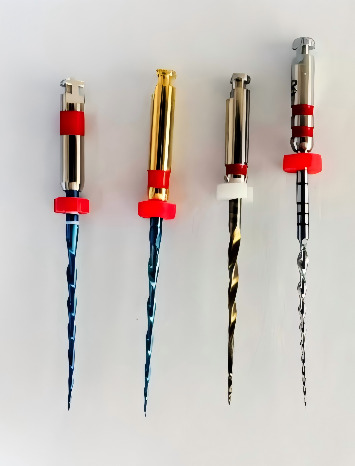
Four single-file Ni–Ti instruments (from left to right: RCB, V-Blue, S-ONE, and One Plex file).

Root canal preparation was conducted using a VDW Silver Reciproc motor (VDW, Munich, Germany) and a SANI motor (EDO-1 Pro, SANI, Chengdu, China) with specific operational parameters for RCB, V-Blue, One Plex, and S-ONE as outlined in Table 1. The appearances of the four Ni–Ti instruments are shown in Figure 2. All procedures were performed by an endodontist with 10 years of clinical experience, in a controlled 37°C water bath, employing a crown-down technique to ensure procedural consistency and standardization. Minimal pressure was applied during preparation, with slow, controlled motions of the Ni–Ti file in the canal. The amplitude of each movement did not exceed 3 mm, and the instrument was withdrawn after three upward and downward strokes, with each file remaining in the canal for no longer than 5 s. Instruments were cleaned with 75% alcohol gauze after each canal preparation. Prior to reinsertion, the instruments were irrigated using a 30-gauge side-vented needle (ChuanHeLeMei, Liaoning, China), with a pull length of less than 2 mm of the working length, followed by 2 mL of distilled water and recanalization with an ISO 15/0.02 K-file (MANI, INC., Japan). A second irrigation of 2-mL distilled water was performed to prevent apical debris blockage. The preparation time for each Ni–Ti file was recorded, excluding irrigation and file-changing intervals. To eliminate instrument fatigue-related bias, each file was used for a single root canal preparation.

### 2.4. Debris Collection and Weighing

Upon completion of root canal preparation, the glass bottle cap and resin root canal were removed, ensuring the canal opening remained vertical to the centrifuge tube and was not in contact with any surfaces. Debris adhering to the apical foramen and below was collected by rinsing the root with 2 mL of distilled water while in the centrifuge tube, ensuring the apical foramen was not directly disturbed to avoid experimental deviations. After debris collection, additional liquid was added to the centrifuge tube up to a total of 10 mL. The tube was then centrifuged (Cence, Changsha, China) at 9000 rpm for 5 min, after which the supernatant was discarded, leaving a small volume of liquid and debris at the bottom. The centrifuge tubes were stored at −20°C (Haier, Qingdao, China) for 6 h, followed by lyophilization for 12 h using a freeze dryer. The weight of each tube was measured using a precision balance (average weight three times), with the postlyophilization weight recorded. The weight difference between the pre- and postdrying measurements was used to determine the weight of the apically extruded debris.

### 2.5. Image Processing

**Figure 3:**
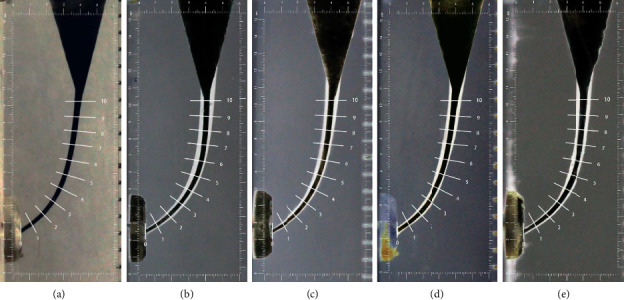
Pre- and postpreparation images of the simulated curved resin canals. (a) Initial shape of root canals before preparation. Representative images of the canals instrumented using (b) RCB; (c) V-Blue; (d) One Plex; (e) S-ONE.

Following root canal preparation, ultrasonic oscillation was applied for 20 min, and absorbent paper points were used to dry the root canal. Black ink was then injected into the canal, and postoperative images were captured using a fixed photographic system to document the simulated curved root canal. The images were processed using the following procedure:

1. In Photoshop CC 2019, a box measuring 15.5 mm × 6 mm was drawn, and 11 measurement lines (Levels 0–10) were set based on the root canal's trajectory. The first measurement line was placed at the apical foramen (Level 0), and the last measurement line was 10 mm from the apical foramen (Level 10), with intervals of 1 mm between the lines.
2. All images were imported into Photoshop CC 2019, where brightness adjustments were made. The images of postpreparation were inverted. All images were then saved in JPEG format.
3. The pre- and postpreparation images of the root canal were aligned with the measurement diagram created in Step 1 to generate a composite image, as shown in Figure 3.

**Figure 4:**
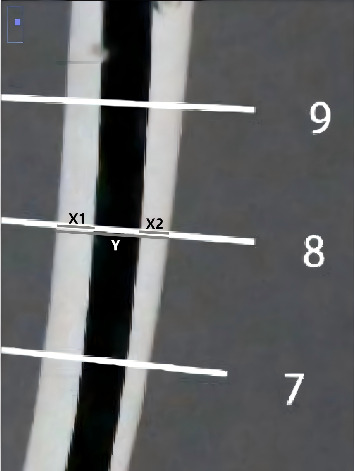
Schematic diagram of Image J software measurement.

The composite images were analyzed and measured using ImageJ software, as shown in Figure 4. Calculations were performed using the following formulas to qualitatively and quantitatively evaluate the shaping ability of four single Ni–Ti files:

1. Resin removal amount on the inner side of the root canal: X1;
2. Resin removal amount on the outer side of the root canal: X2;
3. The final width of the prepared root canal: Y;
4. Total amount of resin removal: X1 + X2;
5. Centering ratio: (X1 − X2)/Y

The centering ability refers to the ability of the Ni–Ti instrument to remain centered during root canal preparation, evaluated by calculating the centering ratio. When X1 and X2 are closer to the canal center, the centering ratio approaches 0. Therefore, the closer the centering ratio was to zero, the better the centering ability of the Ni–Ti instrument.

### 2.6. Statistical Analysis

Statistical analysis was performed using IBM SPSS Statistics 27.0 software (SPSS Inc., Chicago, USA). The Shapiro–Wilk test was used to verify the normality of each dataset. If the data were normally distributed and met the assumption of homogeneity of variance, a one-way analysis of variance (ANOVA) was conducted for multiple group comparisons, followed by Duncan's test for pairwise comparisons. In cases where the assumption of homogeneity of variance was not met, Tamhane's test was applied for multiple comparisons. A *p* value of < 0.05 was considered statistically significant.

## 3. Results

### 3.1. Deformation and Separation of Instruments

**Figure 5:**
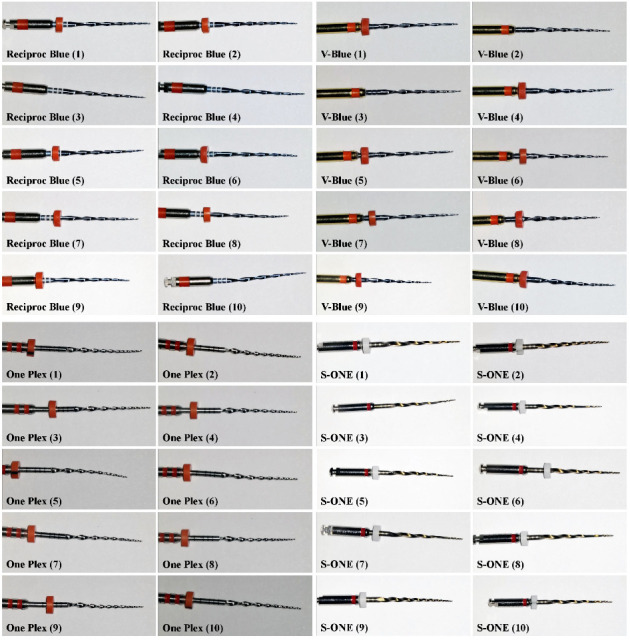
Microscopic examination revealed no deformation in the four groups of instruments.

During this experiment, none of the four single Ni–Ti files experienced separation after preparing an average of one resin-simulated curved root canal. Under the microscope (OPMI S7, Carl Zeiss, Oberkochen, Germany), no significant deformation of the instruments was observed, as shown in Figure 5.

#### 3.1.1. Root Canal Preparation Time

**Figure 6:**
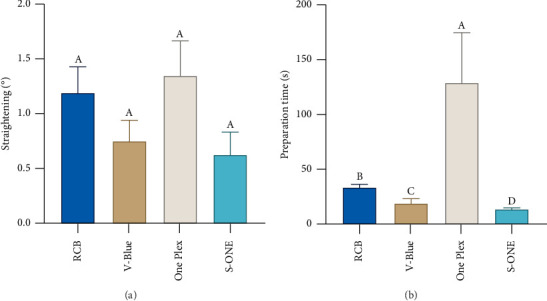
(a) Straightening degree of curved root canals after preparation with the different instruments. (b) Preparation time with the different instruments. Groups marked by different letters are significantly different from each other (*p* < 0.05) whereas marked with the same letter are not significantly different (*p* > 0.05).

Among the groups, the One Plex group had the longest preparation time, while the S-ONE group had the shortest preparation time. There were statistically significant differences between the four groups (*p* < 0.001), as shown in Figure 6.

#### 3.1.2. Change in Curvature of Root Canals

The curvature of the root canals was measured before and after root canal preparation according to the Schneider method [19], and the differences in curvature were calculated. The changes in curvature before and after preparation for the RCB, V-Blue, One Plex, and S-ONE were 1.196 ± 0.752°, 0.760 ± 0.588°, 1.355 ± 0.993°, and 0.633 ± 0.660°, with no statistically significant differences among the four groups (*p* > 0.05), as shown in Figure 6.

#### 3.1.3. Amount of Resin Removal on the Inside and Outside of the Root Canal (X1 and X2)

For the resin removal amount on the inner side of the root canal (X1) in the apical region (Levels 0–4): at Level 0, V-Blue removed the most, while RCB the least (*p* < 0.05); at Level 1, V-Blue removed the most, while One Plex the least (*p* < 0.05); and at Level 2, RCB removed the most, while One Plex the least (*p* < 0.05).

For the resin removal amount on the outer side of the root canal (X2) in the apical region (Levels 0–4): at Level 0, One Plex removed the most (*p* < 0.01); at Level 2, RCB and V-Blue exhibited higher removal amount compared with S-ONE (*p* < 0.05); at Level 3, RCB and V-Blue exhibited higher removal amount compared with One Plex and S-ONE (*p* < 0.01); and at Level 4, both RCB, V-Blue, and S-ONE removed more resin than One Plex (*p* < 0.05).

For the resin removal amount on the inner side of the root canal (X1) in the middle and upper segments (Levels 5–10): at Levels 6–9, RCB removed the most, while One Plex the least (*p* < 0.05).

For the resin removal amount on the outer side of the root canal (X2) in the middle and upper segments (Levels 5–10): at Level 5, V-Blue and S-ONE removed more than One Plex (*p* < 0.05) and at Level 6, S-ONE removed the most, while RCB the least (*p* < 0.05) (Table 2).

#### 3.1.4. Total Amount of Resin Removal

The total amount of resin removal refers to the combined amount of resin removed from both the inner and outer sides of the root canal (X1 + X2)

**Figure 7:**
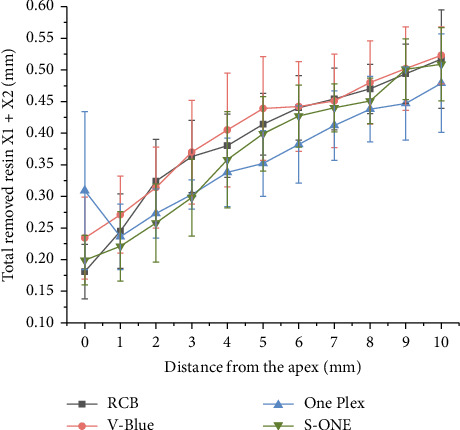
Total removed resin X1 + X2 (mm) at the different levels after root canal preparation.

Based on the data from Table 3 and Figure 7, significant differences were observed among the four groups of Ni–Ti instruments at Levels 0, 2, 3, and 5 (*p* < 0.05). At Level 0, One Plex exhibited the highest total resin removal (*p* < 0.05). At Level 2, RCB showed the greatest removal total, while S-ONE had the least (*p* < 0.05). At Level 3, the removal totals for RCB and V-Blue were significantly higher than those of One Plex and S-ONE (*p* < 0.05). At Level 4, V-Blue removed the most resin whereas One Plex removed the least (*p* < 0.05). At Levels 5 and 6, RCB and V-Blue exhibited higher removal totals compared with One Plex (*p* < 0.05). At Level 9, both V-Blue and S-ONE removed more resin than One Plex (*p* < 0.05).

#### 3.1.5. Centering Ability

**Figure 8:**
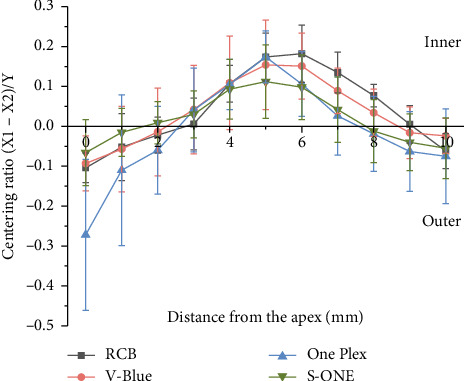
Centering ratio (X1 − X2)/Y at the different levels after root canal preparation (the negative value represents deviation toward the outer wall of the root canal, and the positive value represents deviation toward the inner wall of the root canal).

The centering ability test results for the four groups across 11 observation points are presented in Table 4 and Figure 8. The closer the centering ratio was to zero, the better the centering ability of the Ni–Ti instrument. A negative ratio reflects deviation toward the outer wall of the root canal, while a positive ratio indicates deviation toward the inner wall.

At the apical foramen (Level 0), all four groups showed deviation towards the outer wall of the root canal, with One Plex exhibiting a significantly greater deviation than the other three groups (*p* < 0.05).

At Levels 3–7, all groups shifted toward the inner side of the root canal. At Levels 1, 2, and 5, the S-ONE group demonstrated superior centering ability. At Level 3, the RCB group showed better centering ability. At Level 6, the deviation in the RCB group was significantly greater than that in the One Plex and S-ONE groups (*p* < 0.05).

At Levels 7–8, the RCB group displayed a significantly greater deviation than the One Plex and S-ONE groups (*p* < 0.05). At Level 8, both the One Plex and S-ONE groups deviated laterally, while at Level 9, only the RCB group deviated medially, with the other three groups deviating laterally. The deviation of the One Plex group was significantly greater than that of the RCB group (*p* < 0.05). At Level 10, all four groups deviated laterally, and no statistically significant differences were observed between the groups (*p* > 0.05).

#### 3.1.6. Amount of Apically Extruded Debris

**Figure 9:**
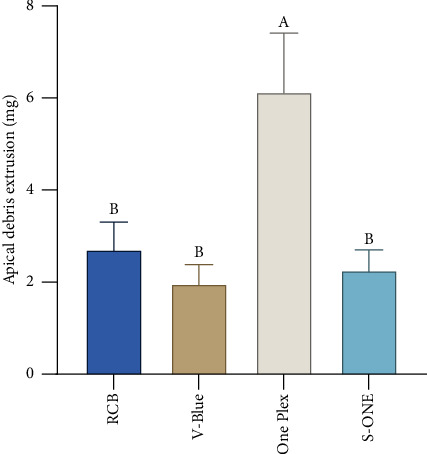
Apical debris extrusion values with the different instruments. Groups marked by different letters are significantly different from each other (*p* < 0.05) whereas marked with the same letter are not significantly different (*p* > 0.05).

A comparison of the amounts of debris extruded beyond the apical foramen across 4 groups is provided in Figure 9. The amount of apical debris extrusion in the One Plex group was greater than that in the RCB group, V-Blue group, and S-ONE group, with a statistically significant difference (*p* < 0.01). There was no significant difference in the amount of apical debris extrusion between the RCB, V-Blue, and S-ONE groups (*p* > 0.05).

## 4. Discussion

This study evaluates shaping ability across three key dimensions: root canal preparation time, changes in curvature, and centering ability. In the current study, while simulated canals in resin blocks standardize canal morphology—such as working length, curvature angle, curvature radius, and diameter—this approach has limitations. It does not offer a three-dimensional view of the canals and their cross-sections, and the mechanical properties of resin differ from those of human teeth. However, because the experimental conditions are consistent across instruments, results obtained from simulated canals in resin blocks are likely to be relevant for natural teeth. Although microcomputed tomography imaging represents the most accurate way for evaluating canal transportation induced by Ni–Ti instruments [22, 23], the superposition technique remains a widely accepted method for assessing shaping efficacy in simulated root canals [24, 25].

### 4.1. Shaping Ability of Four Ni–Ti Instruments

In this study, all procedures were performed by a single operator under identical conditions, with preparation time defined as the duration of the instrument's movement within the root canal. The results revealed statistically significant differences in preparation time among the four types of Ni–Ti instruments. The preparation time ranked as follows: One Plex > RCB > V-Blue > S-ONE, and shorter preparation times corresponded to higher preparation efficiency. Although One Plex file was made from CM-wire, it was not subjected to thermal treatment, lacks an oxide layer on its surface, and features a variable thread cross-section design. This design transitions from a double-edged cross-section at the 0–6 mm tip to an S-shaped cross-section in the 6–16 mm section. The tip is noncutting, serving only as a guide, which diminishes One Plex's cutting efficiency and prolongs the preparation time. In contrast, new thermal processing of CM-wire alloys, involving repeated heating and cooling, forms a blue or golden oxide layer on the surface of Ni–Ti instruments, thereby enhancing cutting efficiency [26–28]. The presence of an oxide layer on the surface of RCB, V-Blue, and S-ONE resulted in shorter preparation times compared with One Plex.

Pre- and postpreparation, all four instrument groups exhibited a tendency to straighten curved root canals; however, no significant differences in canal curvature changes were observed, consistent with previous studies [16]. The majority of prior investigations have excluded postpreparation canal diameter in centering ratio calculations, relying exclusively on canal transportation measurements. The centering ratio formula employed in this study, (X1 − X2)/Y, demonstrates enhanced precision compared with approaches quantifying resin removal volume alone [29]. The centering ability of the four instruments varied according to their distinct design features. In the upper canal segment (Levels 9–10), all instruments demonstrated minimal deviation, indicating excellent centering ability. In the initial curvature segment (Levels 6–8), both S-ONE and One Plex exhibited superior centering ability compared with RCB and V-Blue. At the point of greatest curvature (Levels 5–6), the instruments tended to cut more on the canal's inner wall than the outer, resulting in the poorest centering ability at this location. In the apical segment (Levels 0–3), all files exhibited a tendency to deviate toward the outer canal wall, with One Plex showing the most pronounced outward deviation relative to the other three files. This may be due to the lack of cutting ability in its tip, which functions primarily as a guide in the middle-upper segment, leading to greater deviation. Throughout the preparation process, S-ONE maintained superior centering ability, particularly in the apical segment (Levels 0–3), demonstrating strong conformance to the canal shape. Both RCB and V-Blue, constructed from Blue-wire alloy and featuring double S-shaped cross-sections, displayed similar centering abilities; however, V-Blue caused less deviation than RCB, potentially due to its MaxTech 6.0 Ni–Ti alloy technology, which enhances flexibility and resistance to cyclic fatigue. In a meta-analysis on the centering ability of Ni–Ti instruments, Gundappa et al. [30] identified taper as a key factor contributing to root canal deviation. Instruments with smaller tapers offer improved flexibility, better preservation of the root canal's original morphology, reduced canal transportation, and less dentin removal. This explains why the two instruments with smaller tapers (S-ONE and One Plex) resulted in a lower total amount of resin removal from the root canal in this study.

### 4.2. Amount of Apically Extruded Debris

In the mechanical–chemical preparation of root canals, the use of different instruments and techniques often leads to the extrusion of dentin debris, irrigating solutions, and pulp tissue through the apical foramen [31, 32]. The expulsion of dentin debris and infectious substances, which may contain microorganisms, can disrupt the apical region's original environment, triggering the body's immune response and leading to periapical tissue inflammation [33]. In prior research on apical debris extrusion, methods such as oven drying at temperatures between 70°C and 150°C or air-drying for 5–14 days have been employed [34]. These methods, however, are time-consuming and susceptible to environmental fluctuations in temperature and humidity, which can negatively impact the experiment's standardization. Moisture in the air may adhere to the debris, further complicating the assessment [35]. In this study, a freeze-drying technique was employed to standardize debris collection. The samples were frozen, and moisture was removed via freeze-drying using low-temperature sublimation in a high-vacuum environment. Throughout the process, the centrifuge tubes remained closed, preventing exposure to temperature or humidity variations, which ensured the integrity of the collected debris and maintained the consistency of the experimental condition.

The results of this experimental study demonstrate that all four types of single-file Ni–Ti instruments utilized in this research lead to debris extrusion through the apical foramen, aligning with the consensus in the literature that all Ni–Ti systems cause apical debris extrusion [36–38]. Notably, the continuous rotational single-file instrument, One Plex, resulted in greater apical debris extrusion, a finding that contradicts the conclusions of Ahmad et al. [39–41], who suggested that reciprocating instruments may cause more apical debris extrusion than continuous rotational instruments. This discrepancy can likely be attributed to the design characteristics of One Plex. The One Plex file lacks cutting ability at the root tip within the 0–6 mm range and functions primarily as a guide, which results in suboptimal resin removal capability. This limitation necessitates frequent rinsing during preparation and the use of hand instruments for refiling, potentially leading to increased apical debris extrusion. In contrast, no statistically significant difference in the volume of apical debris expelled was observed among the three reciprocating single-file systems—RCB, V-Blue, and S-ONE—likely due to the superior debris removal capability of these instruments. After the root canal preparation, substantial amounts of debris were observed adhering to the grooves of these three instruments under microscopic examination. The double S-shaped cross-sectional design of both RCB and V-Blue enhanced debris clearance, facilitating the removal of coronal debris. Consistent with findings from researchers such as Predin Djuric et al. [42], it can be inferred that the increased root canal volume, resulting from a greater taper and cross-sectional diameter, contributes to the production of more debris during the preparation process. However, the isolated reciprocating movement does not appear to induce additional apical debris extrusion. Therefore, it can be concluded that apical debris extrusion is influenced by multiple factors, including the movement mode of the Ni–Ti system, as well as the taper, cross-sectional shape, and groove depth of the instrument. These factors collectively affect both the formation of apical debris and its subsequent removal from the root canal.

## 5. Conclusions

According to the study, S-ONE demonstrated the best centering ability and significantly reduced instrumentation time compared with the other three file systems. In contrast, One Plex produced the highest amount of apical debris extrusion and exhibited transportation at the apical foramen, rendering it unsuitable for preparing severely curved canals. At the midcoronal level (6–8 mm from the apex), RCB exhibited excessive removal of the inner canal wall relative to S-ONE and One Plex.

However, this study has certain limitations, as there is a discrepancy in mechanical properties between the resin and extracted teeth. Future work on this topic includes incorporating extracted human tooth models for further comparison. In addition, three-dimensional finite element analysis could be employed to further examine the influence of Ni–Ti instrument taper and cross-sectional morphology variations on shaping ability. Nondestructive optical detection methods, such as Optical Coherence Tomography (OCT) and Photoacoustic Imaging (PAI), may also be allowed for noninvasive evaluation of dental treatments on patients.

## Figures and Tables

**Table 1:** Parameters for each group of Ni–Ti instruments.

Ni–Ti instruments	Size	Alloy material	Cross-sectional shape	Motion mode
RCB	0.08/25	Blue-wire	S-shaped	Reciprocating motion
V-Blue	0.08/25	Blue-wire	S-shaped	Reciprocating motion
One Plex	0.06/25	CM-wire	Double-edged (0–6 mm) S-shaped (6–16 mm)	Continuous rotary motion
S-ONE	0.04/25	Gold-wire	Triangled	Reciprocating motion

**Table 2:** Means of removed resin (mm) and standard deviations (SDs) at the different measurement points after root canal preparation.

Groups (*n* = 10)	Measure points (mm from the apex) Inner canal wall (X1)											Measure points (mm from the apex) Outer canal wall (X2)										
	0	1	2	3	4	5	6	7	8	9	10	0	1	2	3	4	5	6	7	8	9	10
*RCB*																						
Mean	0.07^b^	0.11^ab^	0.16^a^	0.18^a^	0.22^a^	0.26^a^	0.28^a^	0.28^a^	0.27^a^	0.25^a^	0.23^a^	0.10^b^	0.13^a^	0.17^a^	0.18^a^	0.16^a^	0.15^ab^	0.15^b^	0.17^a^	0.21^a^	0.25^a^	0.30^a^
SD	0.02	0.03	0.03	0.03	0.03	0.03	0.03	0.03	0.03	0.03	0.03	0.02	0.04	0.04	0.04	0.03	0.03	0.02	0.02	0.02	0.03	0.04

*VB*																						
Mean	0.10^a^	0.12^a^	0.15^ab^	0.19^a^	0.23^a^	0.27^a^	0.27^a^	0.25^ab^	0.25^ab^	0.24^a^	0.25^a^	0.14^b^	0.15^a^	0.16^a^	0.18^a^	0.17^a^	0.17^a^	0.17^ab^	0.2^a^	0.23^a^	0.26^a^	0.27^a^
SD	0.03	0.03	0.03	0.05	0.07	0.07	0.06	0.05	0.04	0.03	0.03	0.04	0.05	0.05	0.04	0.03	0.04	0.03	0.04	0.04	0.05	0.03

*OP*																						
Mean	0.08^ab^	0.09^b^	0.12^b^	0.16^a^	0.2^a^	0.23^a^	0.23^b^	0.22^c^	0.21^c^	0.2^b^	0.21^a^	0.25^a^	0.14^a^	0.15^ab^	0.14^b^	0.13^b^	0.12^b^	0.16^ab^	0.2^a^	0.23^a^	0.25^a^	0.27^a^
SD	0.03	0.03	0.04	0.04	0.03	0.03	0.04	0.04	0.03	0.04	0.06	0.13	0.07	0.02	0.02	0.01	0.04	0.04	0.05	0.05	0.06	0.07

*SO*																						
Mean	0.09^ab^	0.11^ab^	0.13^ab^	0.16^a^	0.22^a^	0.24^a^	0.24^ab^	0.24^bc^	0.22^bc^	0.23^ab^	0.23^a^	0.11^b^	0.11^a^	0.13^b^	0.13^b^	0.16^a^	0.16^a^	0.18^a^	0.21^a^	0.23^a^	0.27^a^	0.28^a^
SD	0.02	0.03	0.03	0.04	0.05	0.05	0.04	0.04	0.04	0.04	0.04	0.03	0.03	0.03	0.01	0.03	0.03	0.02	0.03	0.03	0.03	0.05

*p* value							^∗^	^∗∗^	^∗∗^	^∗^		^∗∗^			^∗∗^	^∗^	^∗^					

*Note:* RCB: Reciproc Blue.

Abbreviations: OP, One Plex; SO, S-ONE; VB, V-Blue.

^∗^ *p* < 0.05.

^∗∗^ *p* < 0.01 (ANOVA test).

^a,b^Groups marked by different letters are significantly different from each other (*p* < 0.05) whereas marked with the same letter are not significantly different (*p* > 0.05).

**Table 3:** Means of total removed resin (mm) and standard deviations (SDs) at the different measurement points after root canal preparation.

Groups (*n* = 10)	Measure points (mm from the apex) X1 + X2										
	0	1	2	3	4	5	6	7	8	9	10
*RCB*											
Mean	0.181^b^	0.245^a^	0.324^a^	0.363^a^	0.38^ab^	0.414^a^	0.440^a^	0.454^a^	0.470^a^	0.494^ab^	0.517^a^
SD	0.043	0.059	0.066	0.057	0.05	0.049	0.051	0.049	0.039	0.047	0.078

*V-Blue*											
Mean	0.234^b^	0.271^a^	0.314^ab^	0.370^a^	0.405^a^	0.439^a^	0.442^a^	0.451^a^	0.480^a^	0.502^a^	0.523^a^
SD	0.065	0.061	0.064	0.082	0.09	0.082	0.071	0.074	0.066	0.066	0.045

*One Plex*											
Mean	0.309^a^	0.236^a^	0.273^ab^	0.303^b^	0.338^b^	0.352^b^	0.382^b^	0.412^a^	0.438^a^	0.447^b^	0.479^a^
SD	0.125	0.052	0.039	0.023	0.054	0.052	0.061	0.055	0.052	0.058	0.078

*S-ONE*											
Mean	0.199^b^	0.221^a^	0.258^b^	0.298^b^	0.358^ab^	0.399^ab^	0.427^ab^	0.440^a^	0.451^a^	0.501^a^	0.509^a^
SD	0.039	0.055	0.062	0.061	0.076	0.059	0.049	0.038	0.036	0.048	0.058

*p*	^∗^		^∗^	^∗^		^∗^					

^∗^ *p* < 0.05.

^a,b^Groups marked by different letters are significantly different from each other (*p* < 0.05) whereas marked with the same letter are not significantly different (*p* > 0.05).

**Table 4:** Means of centering ratio and standard deviations (SDs) at the different measurement points after root canal preparation.

Groups (*n* = 10)	Measure points (mm from the apex) (X1 − X2)/Y										
	0	1	2	3	4	5	6	7	8	9	10
*RCB*											
Mean	−0.104^a^	−0.052^a^	−0.022^a^	0.006^a^	0.107^a^	0.174^a^	0.182^a^	0.135^a^	0.077^a^	0.005^a^	−0.062^a^
SD	0.037	0.084	0.045	0.065	0.046	0.060	0.072	0.051	0.028	0.047	0.044

*V-Blue*											
Mean	−0.093^a^	−0.057^a^	−0.014^a^	0.042^a^	0.109^a^	0.154^a^	0.151^ab^	0.089^ab^	0.034^ab^	−0.016^ab^	−0.024^a^
SD	0.069	0.107	0.11	0.111	0.117	0.112	0.083	0.057	0.059	0.065	0.044

*One Plex*											
Mean	−0.272^b^	−0.110^a^	−0.060^a^	0.041^a^	0.105^a^	0.174^a^	0.106^b^	0.026^b^	−0.019^b^	−0.063^b^	−0.075^a^
SD	0.189	0.189	0.11	0.105	0.063	0.065	0.081	0.098	0.094	0.1	0.119

*S-ONE*											
Mean	−0.066^a^	−0.015^a^	0.009^a^	0.030^a^	0.093^a^	0.112^a^	0.098^b^	0.042^b^	−0.012^b^	−0.04^ab^	−0.055^a^
SD	0.083	0.06	0.053	0.059	0.075	0.092	0.081	0.082	0.079	0.071	0.076

*p* value	^∗^							^∗∗^	^∗∗^		

*Note:* The negative value represents deviation toward the outer wall of the root canal, and the positive value represents deviation toward the inner wall of the root canal.

^∗^ *p* < 0.05.

^∗∗^ *p* < 0.01.

^a,b^Groups marked by different letters are significantly different from each other (*p* < 0.05) whereas marked with the same letter are not significantly different (*p* > 0.05).

## Data Availability

The data that support the findings of this study are available from the corresponding author upon reasonable request.

## References

[1] Mirza M. B., Gufran K., Alhabib O. (2022). CBCT Based Study to Analyze and Classify Root Canal Morphology of Maxillary Molars: A Retrospective Study. *European Review for Medical and Pharmacological Sciences*.

[2] Oliveira Neto R. S., Alcalde M. P., Titato P. C. G. (2025). Shaping Ability and Cyclic Fatigue Resistance Between Genius ProFlex, ZenFlex, and TruNatomy Rotary Systems: An Experimental Study. *Restorative Dentistry and Endodontics*.

[3] Basmadjian-Charles C. L., Farge P., Bourgeois D. M., Lebrun T. (2002). Factors Influencing the Long-Term Results of Endodontic Treatment: A Review of the Literature. *International Dental Journal*.

[4] Copelli F. A., Oda L. Y., Leal R. M. d S., Rodrigues C. T., Duarte M. A. H., Cavenago B. C. (2025). Influence of the Filling Technique on Endodontic Retreatment in Curved Mesial Canals of Mandibular Molars: An In Vitro Study. *Journal of Endodontics*.

[5] Schäfer E., Diez C., Hoppe W., Tepel J. (2002). Roentgenographic Investigation of Frequency and Degree of Canal Curvatures in Human Permanent Teeth. *Journal of Endodontics*.

[6] Siraparapu K. R., Nagamaheshwari X., Patil V. S., Amulya M. R., Adsare S., Prakash K. A. (2024). Comparative Evaluation of Three File Systems for Shaping Curved Canals: An In Vitro Approach. *Journal of Pharmacy and BioAllied Sciences*.

[7] Estrela C., Bueno M. R., Barletta F. B. (2015). Identification of Apical and Cervical Curvature Radius of Human Molars. *Brazilian Dental Journal*.

[8] Weine F. S., Kelly R. F., Lio P. J. (1975). The Effect of Preparation Procedures on Original Canal Shape and on Apical Foramen Shape. *Journal of Endodontics*.

[9] Estrela C., Pecora J. D., Estrela C. R. A. (2017). Common Operative Procedural Errors and Clinical Factors Associated With Root Canal Treatment. *Brazilian Dental Journal*.

[10] Walia H. M., Brantley W. A., Gerstein H. (1988). An Initial Investigation of the Bending and Torsional Properties of Nitinol Root Canal Files. *Journal of Endodontics*.

[11] Kakoienejad M., Najafifard M., Tavassoli-Hojjati S., Hafezi L., Aghaei S. (2025). Comparison of Hand Files, Mtwo, Reciproc, and Gentlefile Rotary Systems Regarding Canal Transportation, Centering Ability, and Obturation Quality of Primary Molars. *Journal of Dentistry*.

[12] Yared G. (2007). Canal Preparation Using Only One Ni‐Ti Rotary Instrument: Preliminary Observations. *International Endodontic Journal*.

[13] Gavini G., Santos M. D., Caldeira C. L. (2018). Nickel-Titanium Instruments in Endodontics: A Concise Review of the State of the Art. *Brazilian Oral Research*.

[14] Zupanc J., Vahdat‐Pajouh N., Schäfer E. (2018). New Thermomechanically Treated NiTi Alloys: A Review. *International Endodontic Journal*.

[15] Shen Y., Hieawy A., Huang X., Wang Z. J., Maezono H., Haapasalo M. (2016). Fatigue Resistance of a 3-Dimensional Conforming Nickel-Titanium Rotary Instrument in Double Curvatures. *Journal of Endodontics*.

[16] Plotino G., Grande N. M., Cotti E., Testarelli L., Gambarini G. (2014). Blue Treatment Enhances Cyclic Fatigue Resistance of Vortex Nickel-Titanium Rotary Files. *Journal of Endodontics*.

[17] Kishore A., Gurtu A., Bansal R., Singhal A., Mohan S., Mehrotra A. (2017). Comparison of Canal Transportation and Centering Ability of Twisted Files, HyFlex Controlled Memory, and Wave One Using Computed Tomography Scan: An In Vitro Study. *Journal of Conservative Dentistry*.

[18] Peters O. A. (2004). Current Challenges and Concepts in the Preparation of Root Canal Systems: A Review. *Journal of Endodontics*.

[19] Peters O. A., Peters C. I., Schönenberger K., Barbakow F. (2003). ProTaper Rotary Root Canal Preparation: Effects of Canal Anatomy on Final Shape Analysed by Micro CT. *International Endodontic Journal*.

[20] Lim K. C., Webber J. (1985). The Validity of Simulated Root Canals for the Investigation of the Prepared Root Canal Shape. *International Endodontic Journal*.

[21] Schneider S. W. (1971). A Comparison of Canal Preparations in Straight and Curved Root Canals. *Oral Surgery, Oral Medicine, Oral Pathology*.

[22] Wei Z., Cui Z., Yan P., Jiang H. (2017). A Comparison of the Shaping Ability of Three Nickel-Titanium Rotary Instruments: A Micro-Computed Tomography Study via a Contrast Radiopaque Technique In Vitro. *BMC Oral Health*.

[23] Wu H., Peng C., Bai Y., Hu X., Wang L., Li C. (2015). Shaping Ability of ProTaper Universal, WaveOne and ProTaper Next in Simulated L-Shaped and S-Shaped Root Canals. *BMC Oral Health*.

[24] Burklein S., Schäfer E. (2013). Critical Evaluation of Root Canal Transportation by Instrumentation. *Endodontic Topics*.

[25] Ajuz N. C., Armada L., Goncalves L. S., Debelian G., Siqueira J. F. (2013). Glide Path Preparation in S‐Shaped Canals With Rotary Pathfinding Nickeltitanium Instruments. *Journal of Endodontics*.

[26] Zinelis S., Darabara M., Takase T., Ogane K., Papadimitriou G. D. (2007). The Effect of Thermal Treatment on the Resistance of Nickel‐Titanium Rotary Files in Cyclic Fatigue. *Oral Surgery, Oral Medicine, Oral Pathology, Oral Radiology and Endodontics*.

[27] Shen Y., Zhou H. M., Zheng Y. F., Campbell L., Peng B., Haapasalo M. (2011). Metallurgical Characterization of Controlled Memory Wire Nickel‐Titanium Rotary Instruments. *Journal of Endodontics*.

[28] De-Deus G., Silva E. J., Vieira V. T. (2017). Blue Thermomechanical Treatment Optimizes Fatigue Resistance and Flexibility of the Reciproc Files. *Journal of Endodontics*.

[29] Goldberg M., Dahan S., Machtou P. (2012). Centering Ability and Influence of Experience When Using Wave One Single-File Technique in Simulated Canals. *International Journal of Dentistry*.

[30] Gundappa M., Khoriya S., Mohan R., Bansal R. (2014). Root Canal Centering Ability of Rotary Cutting Nickel Titanium Instruments: A Meta-Analysis. *Journal of Conservative Dentistry*.

[31] Ruiz-Hubard E. E., Gutmann J. L., Wagner M. J. (1987). A Quantitative Assessment of Canal Debris Forced Periapically During Root Canal Instrumentation Using Two Different Techniques. *Journal of Endodontics*.

[32] Seltzer S., Naidorf I. J. (1985). Flare-Ups in Endodontics: I. Etiological Factors. *Journal of Endodontics*.

[33] Karamifar K., Tondari A., Saghiri M. A. (2020). Endodontic Periapical Lesion: An Overview on the Etiology, Diagnosis and Current Treatment Modalities. *European Endodontic Journal*.

[34] Raj R A., Raju I., Varghese J. G., Jeet Singh Birring O., Paul Yacob P. J., Chohan H. (2024). Comparative Evaluation of Debris Expulsion Beyond Apex During Re-Root Canal Treatment by Utilizing Two Re-Treatment Rotary Files and Two Reciprocating Files: An In-Vitro Study. *Cureus*.

[35] Tanalp J., Kaptan F., Sert S., Kayahan B., Bayirl G. (2006). Quantitative Evaluation of the Amount of Apically Extruded Debris Using 3 Different Rotary Instrumentation Systems. *Oral Surgery, Oral Medicine, Oral Pathology, Oral Radiology and Endodontics*.

[36] Borges Á H., Pereira T. M., Porto A. N. (2016). The Influence of Cervical Preflaring on the Amount of Apically Extruded Debris After Root Canal Preparation Using Different Instrumentation Systems. *Journal of Endodontics*.

[37] Bürklein S., Schäfer E. (2012). Apically Extruded Debris With Reciprocating Single-File and Full-Sequence Rotary Instrumentation Systems. *Journal of Endodontics*.

[38] Üstün Y., Çanakçi B. C., Dinçer A. N., Er O., Düzgün S. (2015). Evaluation of Apically Extruded Debris Associated With Several Ni-Ti Systems. *International Endodontic Journal*.

[39] Ahmad M. Z., Sadaf D., MacBain M. M., Merdad K. A. (2022). Effect of Mode of Rotation on Apical Extrusion of Debris With Four Different Single-File Endodontic Instrumentation Systems: Systematic Review and Meta-Analysis. *Australian Endodontic Journal*.

[40] Gummadi A., Panchajanya S., Ashwathnarayana S., Santhosh L., Jaykumar T., Shetty A. (2019). Apical Extrusion of Debris Following the Use of Single-File Rotary/Reciprocating Systems, Combined With Syringe or Ultrasonically-Facilitated Canal Irrigation. *Journal of Conservative Dentistry*.

[41] Ahmad M. Z., Kukiattrakoon B. (2024). Assessment of Debris Extrusion in Curved Canals: An In Vitro Analysis of Various Single‐File Endodontic Instrumentation Systems. *International Journal of Dentistry*.

[42] Predin Djuric N., Van Der Vyver P., Vorster M., Vally Z. I. (2021). Comparison of Apical Debris Extrusion Using Clockwise and Counter‐Clockwise Single‐File Reciprocation of Rotary and Reciprocating Systems. *Australian Endodontic Journal*.

